# Fatal *Toxoplasma gondii* infection in the giant panda

**DOI:** 10.1051/parasite/2015030

**Published:** 2015-10-29

**Authors:** Hongyu Ma, Zedong Wang, Chengdong Wang, Caiwu Li, Feng Wei, Quan Liu

**Affiliations:** 1 College of Life Science, Jilin Agricultural University 2888 Xincheng Street Changchun 130118 Jilin Province China; 2 Key Laboratory of Jilin Province for Zoonosis Prevention and Control, Military Veterinary Institute, Academy of Military Medical Sciences Changchun China; 3 China Conservation and Research Center for the Giant Panda Ya’an China

**Keywords:** *Toxoplasma gondii*, Giant panda, Genotype, PCR

## Abstract

*Toxoplasma gondii* can infect nearly all warm-blooded animals. We report an acute fatal *T. gondii* infection in the endangered giant panda (*Ailuropoda melanoleuca*) in a zoo in China, characterized by acute gastroenteritis and respiratory symptoms. *T. gondii* infection was confirmed by immunological and molecular methods. Multilocus nested PCR-RFLP revealed clonal type I at the *SAG1* and *c29-2* loci, clonal type II at the *SAG2*, *BTUB*, *GRA6*, *c22-8*, and *L358* loci, and clonal type III at the alternative *SAG2* and *SAG3* loci, thus, a potential new genotype of *T. gondii* in the giant panda. Other possible pathogens were not detected. To our knowledge, this is the first report of clinical toxoplasmosis in a giant panda.

## Abstract (fr)

*Toxoplasma gondii* peut infecter presque tous les animaux à sang chaud. Nous rapportons une infection fatale aiguë par *T. gondii* chez le panda géant (*Ailuropoda melanoleuca*), une espèce en danger, dans un zoo en Chine, caractérisée par une gastro-entérite aiguë et des symptômes respiratoires. L’infection par *T. gondii* a été confirmée par des méthodes immunologiques et moléculaires. La PCR-RFLP nichée a révélé le type clonal I aux loci *SAG1* et *c29-2*, le type clonal II aux loci *SAG2*, *BtuB*, *GRA6*, *c22-8* et *L358*, le type clonal III aux loci alternatifs *SAG2* et *SAG3*, et donc un nouveau génotype potentiel de *T. gondii* chez le panda géant. Aucun autre agent pathogène n’a été détecté. À notre connaissance, ceci est le premier signalement de toxoplasmose clinique chez le panda géant.

## Abstract (zh)

弓形虫能感染几乎所有的温血动物。本文报道了一例发生在中国动物园的濒危动物大熊猫急性致死性弓形虫感染病例，其主要临床特征表现为急性胃肠炎和呼吸系统症状。弓形虫感染经免疫学及分子生物学方法确证。多位点巢式PCR限制性片段长度多态性分析表明，感染弓形虫SAG1和c29-2位点为I型，SAG2，BTUB，GRA6，c22-8和L358位点为II型，alternative SAG2和SAG3位点为III型，显示大熊猫感染弓形虫可能为一种新基因型。未检测到可导致性胃肠炎和呼吸系统症状的其它病原体。这是首例大熊猫弓形虫感染病例。

## Introduction

Toxoplasmosis, caused by the obligate intracellular protozoan *Toxoplasma gondii*, is an important zoonosis worldwide. It is a major public health concern, mainly because of congenital disease, infection of immunocompromised patients, and an emerging severe form of acquired toxoplasmosis in immunocompetent patients [1]. The lifecycle of *T. gondii* includes sexual multiplication within cats and asexual multiplication within nearly all warm-blooded animals, including humans [6]. Humans and animals become infected by eating undercooked or raw meat containing cysts, or by ingesting food or water contaminated with sporulated oocysts.

The giant panda (*Ailuropoda melanoleuca*) is an emblematic endangered species and regarded as a national treasure and “living fossil” in China [16]. Its population is estimated at approximately 1600 in the wild, and the captive population is more than 300 [7, 13]. The health of giant pandas has attracted global attention. Here, we report an acute fatal *T. gondii* infection in the giant panda in China.

## Case presentation

In February 2014, a seven-year-old giant panda named Jin Yi was found dead at Zhengzhou Zoo, Henan Province, China. The panda did not eat at noon on February 7. On February 8, the panda was found lying in the room with head buried in the abdomen. Treatment measures included intramuscular administration of cephalosporin and intravenous infusion of glucose. The animal had difficulty breathing overnight and was found dead in the morning of February 9.

**Figure 1.**
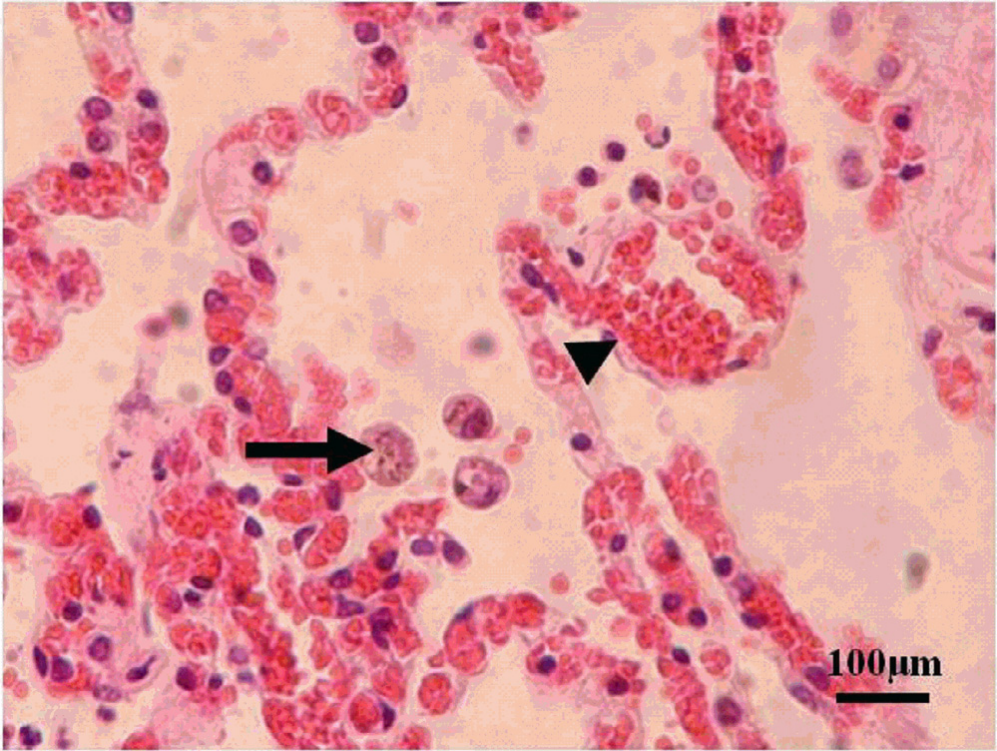
Many macrophages containing *Toxoplasma gondii* tachyzoites (arrow) in the alveoli, and dilated capillaries (arrowhead) in the alveolar wall. Giant panda lung, hematoxylin-eosin stain.

A complete necropsy was conducted. Severe pathologic lesions were found, localized to the gastrointestinal tract and lungs. The gastrointestinal tract contained little or no ingesta, had multifocal mucosal hemorrhage, and dry, hard-packed digesta in the duodenum. Lungs were congested and chyme blocked the respiratory tract. Histologically, macrophages containing *T. gondii* tachyzoites were seen in the alveoli (Fig. 1). Other lesions included congestion in the intestinal lamina propria and submucosa, gastric epithelial necrosis, and sloughing.

**Figure 2.**
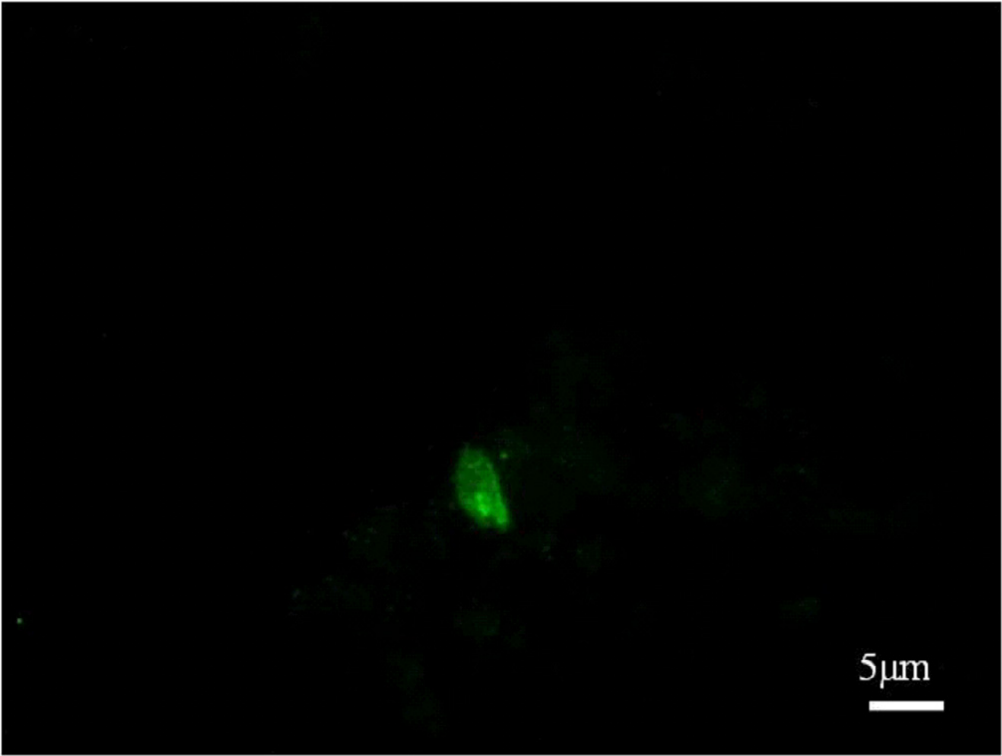
Immunofluorescence assay (IFA) conducted on the frozen tissues using monoclonal antibodies against tachyzoite-specific surface antigen SAG1 showing *Toxoplasma gondii* tachyzoites in the lungs of the giant panda.

Serum and tissue samples were collected for examination of potential pathogens that may cause hemorrhagic gastroenteritis. The animal had an antibody titer for *T. gondii* of 200 by the modified agglutination test [5]. *T. gondii* DNA was detected in the liver, spleen, lungs, kidneys, and small and large intestines by nested PCR targeting the B1 gene [12]. The immunofluorescence assay (IFA) revealed *T. gondii* tachyzoites present in the lung and small intestine tissues (Fig. 2), suggesting acute orally acquired toxoplasmosis in the giant panda, probably occurring 7–10 days before signs.

The positive DNA samples were directly typed by multilocus nested PCR-RFLP (Mn-PCR-RFLP) using 10 genetic markers (*SAG1, SAG2, SAG3, BTUB, GRA6, c22-8, c29-2, L358, PK1,* and *Apico*), and the reference strains, including *GT1, PTG, CTG, MAS, TgCgCa1, TgCatBr5, TgCatBr40, TgCatBr64*, and *TgRsCr1*, were used as positive controls. The results revealed clonal type I at the *SAG1* and *c29-2* loci, clonal type II at the *SAG2*, *BTUB*, *GRA6*, *c22-8*, and *L358* loci, and clonal type III at the alternative *SAG2* and *SAG3* loci, showing a potential new atypical genotype of *T. gondii* in the giant panda. Other potential pathogens, including viruses and bacteria that cause acute gastroenteritis, or respiratory disease, were not detected. These results demonstrated that the giant panda died from acute toxoplasmosis due to a *T. gondii* strain of an atypical genotype.

## Discussion

*Toxoplasma gondii* is considered to be one of the most successful eukaryotic pathogens, based on the number of host species and percentage of animals infected worldwide. The consequences of infection with *T. gondii* are associated with the host species and parasite genotypes. Primary infections in adults are mostly asymptomatic, but severe, acute, disseminated toxoplasmosis can occur in immunocompetent hosts when infected with some isolates [11]. Many *T. gondii* genotypes identified in animals and humans show high genetic diversity of *T. gondii* in China [10]. In addition to the atypical ToxoDB#9, there are several other atypical *T. gondii* genotypes identified in animals and humans in China 1 [2, 17, 14]. Atypical *T. gondii* strains have been shown to cause severe clinical disease in immunocompetent hosts [4, 9].

Despite its taxonomic classification as a carnivore, the giant panda has a diet that is primarily herbivorous, almost exclusively bamboo. The panda still retains decidedly ursine teeth and will eat meat when available [15]. In addition to bamboo, the captive panda is given some formulated biscuits or other dietary supplements. There are a number of stray cats and small rodents in the zoo, and these animals can freely roam in the habitat of the giant panda. The infection may be obtained by consuming food or water contaminated with sporulated oocysts, or by ingestion of rodents infected with *T. gondii* [3]. Treatment of the disease should include pyrimethamine plus sulfadiazine. Avoiding consumption of raw or undercooked meat is the main measure recommended to prevent *T. gondii* infection [8].
